# Computed tomography and [^18^F]-FDG PET imaging provide additional readouts for COVID-19 pathogenesis and therapies evaluation in non-human primates

**DOI:** 10.1016/j.isci.2022.104101

**Published:** 2022-03-17

**Authors:** Thibaut Naninck, Nidhal Kahlaoui, Julien Lemaitre, Pauline Maisonnasse, Antoine De Mori, Quentin Pascal, Vanessa Contreras, Romain Marlin, Francis Relouzat, Benoît Delache, Cécile Hérate, Yoann Aldon, Marit van Gils, Nerea Zabaleta, Raphaël Ho Tsong Fang, Nathalie Bosquet, Rogier W. Sanders, Luk H. Vandenberghe, Catherine Chapon, Roger Le Grand

**Affiliations:** 1 Université Paris-Saclay, Inserm, CEA, Center for Immunology of Viral, Auto-immune, Hematological and Bacterial Diseases (IMVA-HB/IDMIT), Fontenay-aux-Roses & Le Kremlin-Bicêtre, Paris, France; 2 Departments of Medical Microbiology of the Amsterdam UMC, Amsterdam Institute for Infection and Immunity, University of Amsterdam, 1105 Amsterdam, the Netherlands; 3 Grousbeck Gene Therapy Center, Schepens Eye Research Institute, Mass Eye and Ear, Boston, MA 02114, USA; 4 Ocular Genomics Institute, Department of Ophthalmology, Harvard Medical School, Boston, MA 02115, USA; 5 The Broad Institute of Harvard and MIT, Cambridge, MA 02142, USA; 6 Harvard Stem Cell Institute, Harvard University, Cambridge, MA 02138, USA; 7 Department of Microbiology and Immunology, Weill Medical College of Cornell University, New York, NY 10021, USA

**Keywords:** Medical microbiology, Medical imaging, Virology

## Abstract

Non-human primates (NHPs) are particularly relevant as preclinical models for SARS-CoV-2 infection and nuclear imaging may represent a valuable tool for monitoring infection in this species. We investigated the benefit of computed X-ray tomography (CT) and [^18^F]-FDG positron emission tomography (PET) to monitor the early phase of the disease in a large cohort (n = 76) of SARS-CoV-2 infected macaques.

Following infection, animals showed mild COVID-19 symptoms including typical lung lesions. CT scores at the acute phase reflect the heterogeneity of lung burden following infection. Moreover, [^18^F]-FDG PET revealed that FDG uptake was significantly higher in the lungs, nasal cavities, lung-draining lymph nodes, and spleen of NHPs by 5 days postinfection compared to pre-infection levels, indicating early local inflammation. The comparison of CT and PET data from previous COVID-19 treatments or vaccines we tested in NHP, to this large cohort of untreated animals demonstrated the value of *in vivo* imaging in preclinical trials.

## Highlights

• Macaques exposed to SARS-CoV-2 show typical mild COVID-19 lung lesions
• Infected animals have increased metabolism in airways and some lymphatic organs
• PET and CT imaging provide an additional readout to assess therapy efficacy

## Graphical abstract

**Figure:**
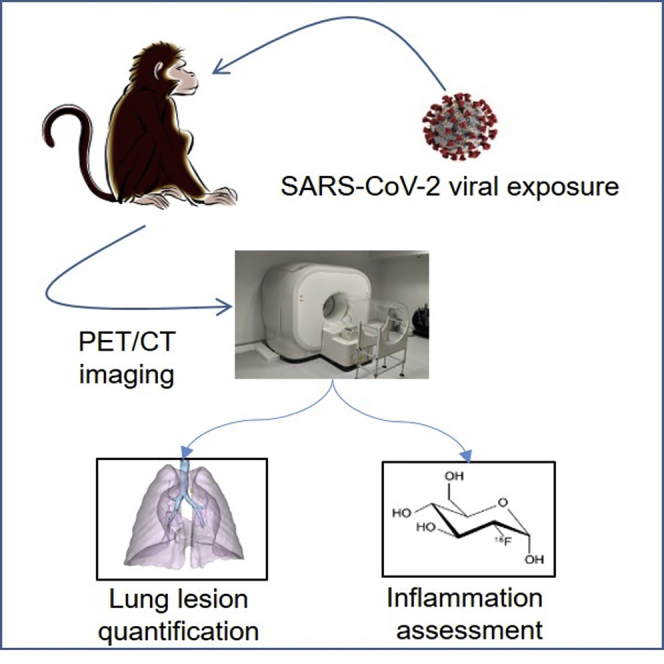

## Introduction

The COVID-19 pandemic is still affecting millions of people worldwide and has been responsible for nearly five million deaths (up to mid-October 2021), according to the WHO (WHO, 2021). From the very beginning of the epidemic, animal models of SARS-CoV-2 infection have been developed to provide rapid and robust approaches for transmission and pathogenesis studies (Muñoz-Fontela et al., 2020). Non-human primates (NHPs) are particularly relevant for studying SARS-CoV-2 infection. Because of their phylogenetic closeness to humans, NHPs express an angiotensin-converting enzyme 2 (ACE-2) receptor that can be used by human viral isolates to enter target cells, without any artificial modification of the host (transgenesis) or the pathogen (inserted mutations and serial passages) (Melin et al., 2020). For the same reasons, NHP models are susceptible to a variety of coronaviruses that affect humans, such as SARS-CoV and MERS-CoV (Lemaitre et al., 2021). SARS-CoV-2 infection studies in NHPs have been reported using various species (mostly rhesus or cynomolgus macaques but also baboons and African green monkeys), with diverse exposure routes and doses (Muñoz-Fontela et al., 2020; Singh et al., 2021; Woolsey et al., 2021). The follow-up of infection in these NHPs can be performed using the same readouts as those used to monitor infection and disease in humans, including viral load quantification, cytokines and antibody responses, and *in vivo* imaging. Because animals used in experimental settings are young adults with no comorbidities, most such NHP models, except in one recent pilot study (Urano et al., 2021), have shown only asymptomatic to mild clinical symptoms following SARS-CoV-2 exposure recapitulating mainly disease and infection profiles in the majority of infected humans, with similar dynamics of viral shedding and antibody response (Munster et al., 2020; Rockx et al., 2020). Chest radiography or CT scan show lung lesions with mainly peripheral opacities, a hallmark of interstitial pneumonia, and acute respiratory distress syndrome (ARDS) (Salguero et al., 2021). Indeed, *in vivo* imaging, especially CT, was reported to be efficient tools, even relative to gold-standard RT-qPCR, for COVID-19 diagnosis during the early outbreak (Ai et al., 2020; Long et al., 2020). NHP models have been used to assess treatments and vaccines efficacy and radiological analysis has been critical for monitoring their impact on the tissue lesions (Maisonnasse et al., 2020; Vogel et al., 2021; Williamson et al., 2020). However, most NHP studies were performed using a small number of animals limiting the power of comparative analysis. No extensive study has been performed thus far to examine longitudinally inflammation or cellular metabolic activation at the whole-body scale in NHPs as it can be approached by [^18^F]-fluorodesoxyglucose ([^18^F]-FDG) positron emission tomography (PET) imaging (Finch et al., 2020; Hartman et al., 2020). Currently, [^18^F]-FDG PET imaging is not recommended as a first choice for the diagnosis of infectious diseases, but several clinical reports have highlighted the added value of this modality with respect to COVID-19 monitoring (Deng et al., 2020; Joob and Wiwanitkit, 2020). As in a number of pilot NHP studies, increased inflammation in the lungs and lymphatic organs has indeed been reported in COVID-19 patient case reports (Dietz et al., 2021; Minamimoto et al., 2020; Setti et al., 2020). Here, we aimed to longitudinally study CT scan images, both qualitatively and quantitatively, in a large cohort of NHP (>70), including both cynomolgus and rhesus macaques following combined intranasal + intra-tracheal exposure to various doses of SARS-CoV-2. We additionally aimed to quantify [^18^F]-FDG uptake at the early stage of the disease in various respiratory and lymphoid organs to better understand early disease progression and host responses and provide additional readouts to evaluate the efficacy of potential new treatments or vaccines.

## Results

### Animal inclusion and disease monitoring

In total, 61 Mauritian cynomolgus macaques (CM) and 15 rhesus macaques (RM) were exposed to the virus (or mock exposed as control) and included in our imaging longitudinal analysis, as described in Tables 1 and S1. Additional imaging data from our previous published studies (Maisonnasse et al., 2020, 2021; Zabaleta et al., 2021) in CM assessing the efficacy of diverse treatments (hydroxychloroquine, n = 5, monoclonal antibody, n = 5) or vaccination (n = 6) against SARS-CoV-2 infection were then used to assess the strength of the imaging readout (Table 2).

**Table 1:** Repartition of untreated and SARS-CoV-2-exposed non-human primates for species, dose, and imaging modality

Species	Dose (PFU)	Imaging modality	N =
Cynomolgus macaque	10^7^	PET-CT	2
Cynomolgus macaque	10^6^	PET-CT	4
Cynomolgus macaque	10^6^	CT	34
Cynomolgus macaque	10^5^	PET-CT	6
Cynomolgus macaque	10^5^	CT	12
Cynomolgus macaque	none	PET-CT	3
Rhesus macaque	10^7^	PET-CT	2
Rhesus macaque	10^6^	PET-CT	1
Rhesus macaque	10^5^	CT	12

PFU: Plaque-forming units.

**Table 2:** Repartition of treated/vaccinated SARS-CoV-2-exposed non-human primates for species, dose, and imaging modality

Species	Dose (PFU)	Treatment/vaccine	Imaging modality	N =
Cynomolgus macaque	10^6^	Hydroxychloroquine	CT	5
Cynomolgus macaque	10^6^	mAb COVA 1-18	CT	5
Cynomolgus macaque	10^5^	AAV-based vaccine	PET-CT	6

PFU: plaque-forming units, mAb: monoclonal antibody.

**Figure 1:**
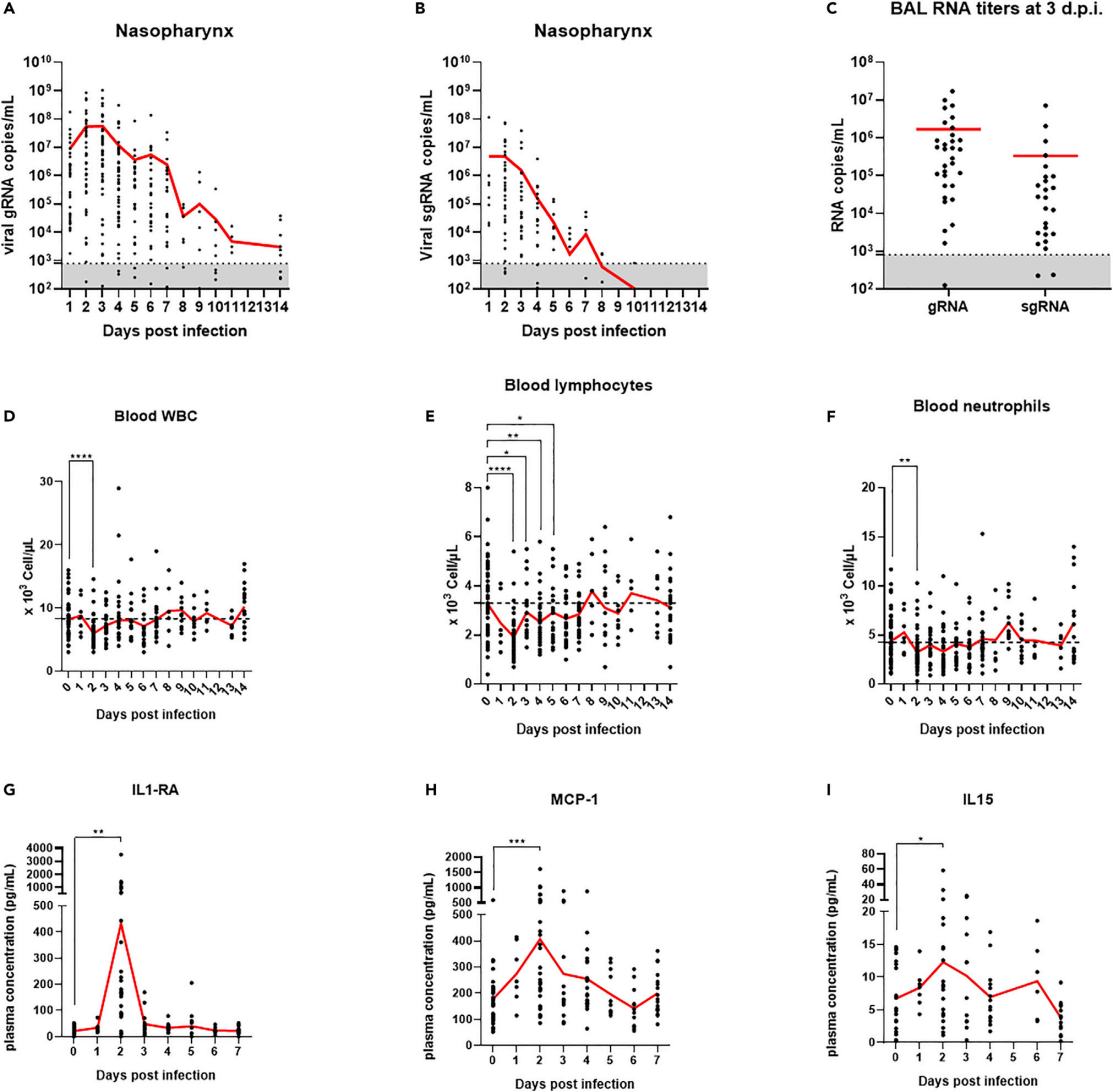
Monitoring of SARS-CoV-2 infection in cynomolgus macaques (A and B) Nasopharynx genomic (A) and subgenomic (B) SARS-CoV-2 RNA copies per milliliter evolution over time following infection. (C) Viral RNA titers evaluated in bronchoalveolar lavages at 3 days postinfection. Dotted lines represent the limit of quantification. (D–F) White blood cells (D), lymphocytes (E), and neutrophils (F) counts in blood over time. (D–F) Dotted lines represent the average baseline values. (G–I) Plasma concentrations of IL1-RA (G), MCP-1 (H) and IL-15 (I) over time. Paired t-tests: ∗∗∗∗: p < 0.0001, ∗∗∗: p < 0.001, ∗∗: p < 0.01, ∗: p < 0.05. Mean values are represented in red.

Virus was detected using genomic and subgenomic RT-qPCR in the upper and lower respiratory tract of infected animals with a peak of detection at 2–3 days postinfection (d.p.i.) followed by a progressive decrease of viral genome in the upper airways over the next two weeks (Figures 1A–1C, S1A, and S1B). Infection of animals also induced a significant (p < 0.0001 for CM and p = 0.013 for RM) transient decrease in blood leukocyte count mostly driven by significant lymphocyte (p < 0.0001 for CM and p = 0.03 for RM) and neutrophil blood counts decrease at 2 days.p.i (Figures 1D–1F, S1C, and S1D). A significant transient increase of some pro-inflammatory cytokines and chemokines such as IL-1RA (p = 0.0025), MCP-1 (p = 0.0004), IL-15 (p = 0.044), and IFNα (p = 0.0024) was also detected at 2–3 days.p.i. in the plasma of infected animals (Figures 1G–1I and S2A). Other pro-inflammatory cytokines such as IL-6 and IFNγ did not present a significant increase over the first two weeks of infection (Figures S2B and S2C).

### Computed tomography scan lung lesion patterns and evaluation

**Figure 2:**
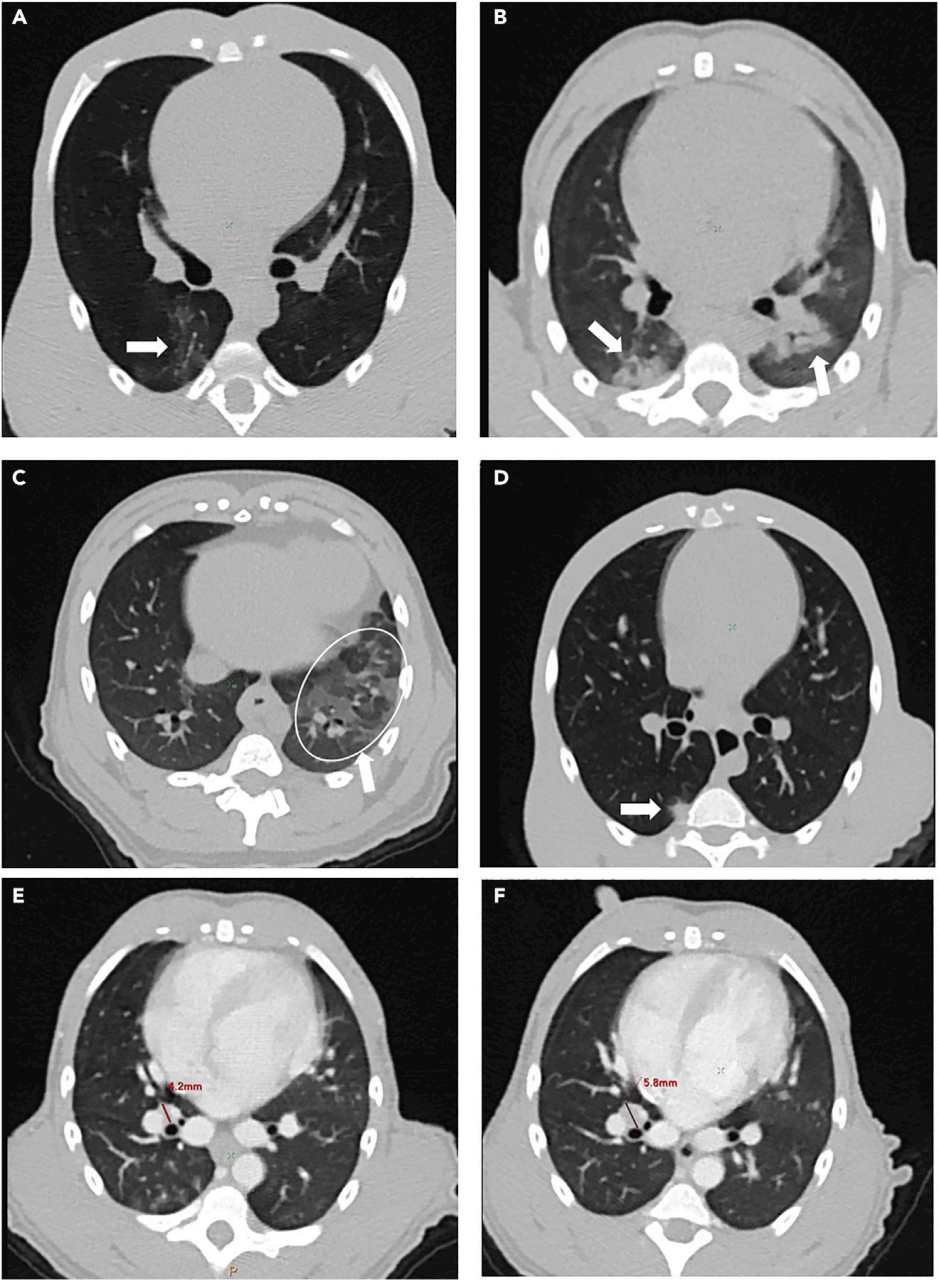
Representative examples of lung lesions observed at 2–3 days.p.i. in transversal chest CT slices in individual SARS-CoV-2-infected cynomolgus macaques (A) Low-density ground-glass opacity (GGO), arrow. (B) Pleural peripheral GGO (left arrow) and peribronchial (right arrow) GGO of higher density. (C) Extended reticulated GGO (circled). (D) Focal pleural consolidation (arrow). (E and F) Tracheobronchial lymph node, showing an increase in volume (E: before exposure, (F) 2 days post-infection (d.p.i.), red lines).

Following SARS-CoV-2 exposure, CM developed lesion features similar to those observed in infected humans (Pan et al., 2020; Shi et al., 2020; Zhu et al., 2020; Zu et al., 2020). This included peripheral ground-glass opacities (GGOs) (Figures 2A–2C, arrows), sometimes associated with intra-lobular septal interstitial thickening or focal consolidations (Figure 1D, arrow), mostly in contact with the pleura. Hypertrophy of thoracic lymph nodes, especially interlobar and subcarinal lymph nodes, could also be observed using an iodine-contrast agent (Representative example in Figures 1E and 1F, the diameter increased from 4.2 mm before exposure to 5.8 mm two days later), as already reported for humans (Albarello et al., 2020). The same lesion features could also be found in SARS-CoV-2-infected RM (Figures S1E and S1F).

We performed semi-quantitative CT scoring to better characterize the lung-lesion burden, as already described for preclinical and clinical SARS-CoV-2-induced lung lesion assessment (Brouwer et al., 2021; Maisonnasse et al., 2020, 2021; Pan et al., 2020; Shi et al., 2020). Such scoring, described in Table S2, reflects the lesion type and extension for each imaging time point.

**Figure 3:**
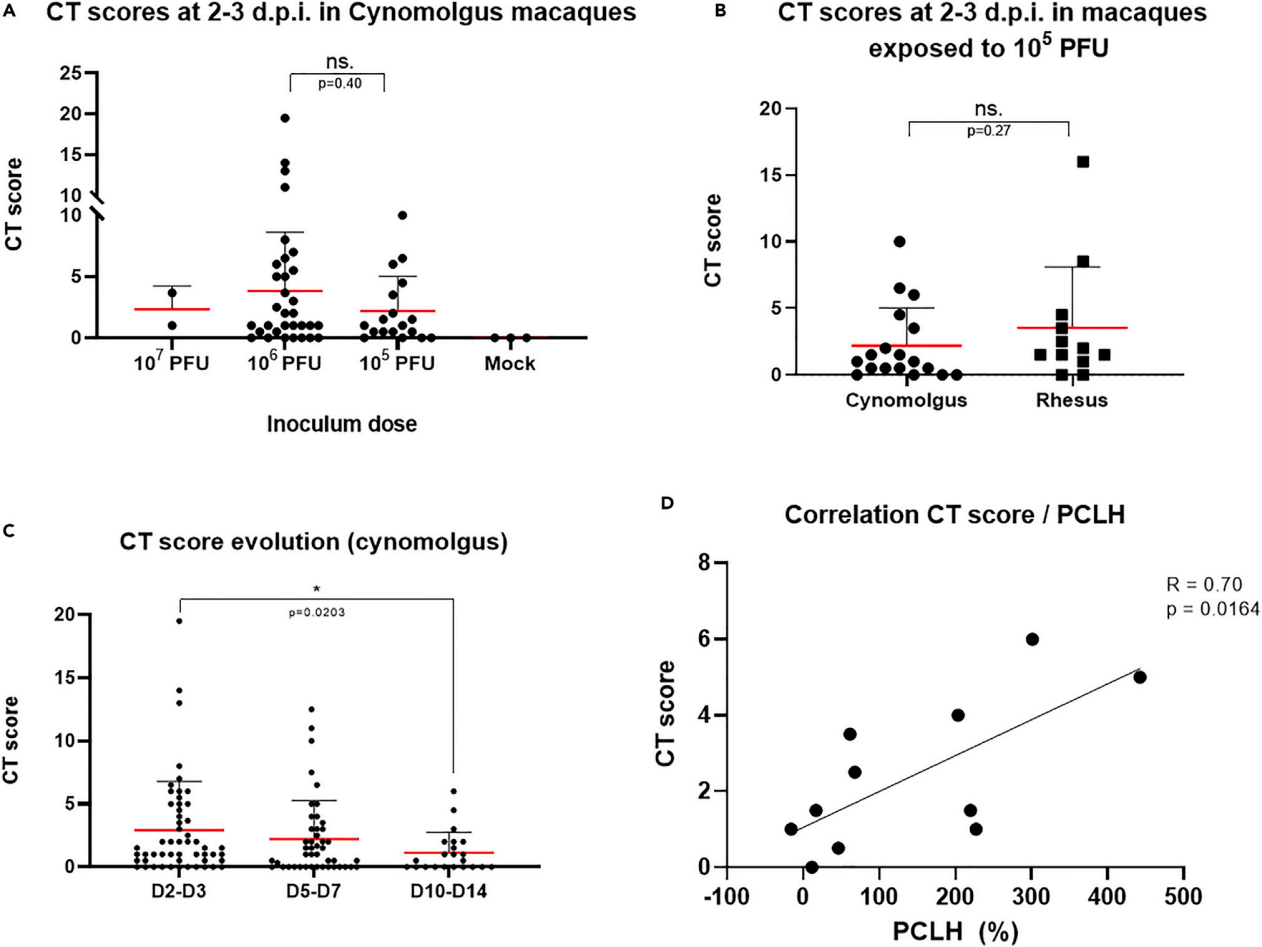
Chest CT scan scoring analysis (A) CT scores at 2–3 days post-infection (d.p.i.) in SARS-CoV-2-exposed cynomolgus macaques according to the inoculum dose. Mock: exposed to PBS. (B) CT scores at 2–3 days.p.i. in SARS-CoV-2 exposed (10^5^ PFU) cynomolgus versus rhesus macaques. (C) Evolution of the CT score between 2 and 3 days.p.i. and 10–14 days.p.i. for exposed cynomolgus macaques (∗: p = 0.0203). (D) Correlation between CT scores and the percentage change in lung hyperdensity (PCLH) for n = 12 cynomolgus macaques; linear regression (R = 0.70, p = 0.016). ns: non statistically significant, Mann–Whitney unpaired (A, B) or paired (C) t-tests. PFU: plaque-forming units. Mean values are represented in red with associated SD.

None of the three mock-infected animals showed lung lesions (CT score = 0). The dose of viral exposure did not appear to affect the CT scores. We observed no statistical difference during the acute phase of infection (2–3 days post-infection (d.p.i.)) between animals exposed to 10^5^ PFU and those exposed to 10^6^ PFU (p = 0.40) for CM (Figure 3A). The two animals exposed to 10^7^ PFU also showed similar CT scores. Infected animals showed a broad range of lung burden (Figure 3A). Most (n = 39/52, 75%) had mild lung lesions (CT scores <5), but a few showed larger lesions (extended GGOs and/or CPP). There was no statistical difference (p = 0.27) concerning the CT scores at 2–3 days.p.i. between cynomolgus and rhesus macaques exposed to 10^5^ PFU of SARS-CoV-2 (Figure 3B). Overall, NHP imaging CT data reflects the diversity of human cases, with mostly mild to moderate cases (Oran and Topol, 2021).

Based on these results, all CM CT scores at 2–3 days.p.i. were pooled, regardless of the initial dose of exposure for the next set of analyses. Additional longitudinal follow-up of 28 CM was performed to assess the progression of the lung burden. We observed a progressive decrease in lung lesions over time from 2 to 3 days.p.i. and reached statistical significance before the end of the second-week post-exposure (p = 0.02) (Figures 3C, S3, and S4).

We also performed quantitative percentage change in lung hyperdensity (PCLH) (Wei et al., 2013) analysis for a subset of CM (n = 11) to address semi-quantitative/operator-dependent issues with the CT scores. We found a significant linear correlation (r = 0.70, p = 0.016) between PCLH and CT scores in this analysis, validating the CT scoring accuracy (Figure 3D).

### Monitoring of metabolic activity in key organs of infected cynomolgus macaques by [^18^F]-FDG PET-CT

**Figure 4:**
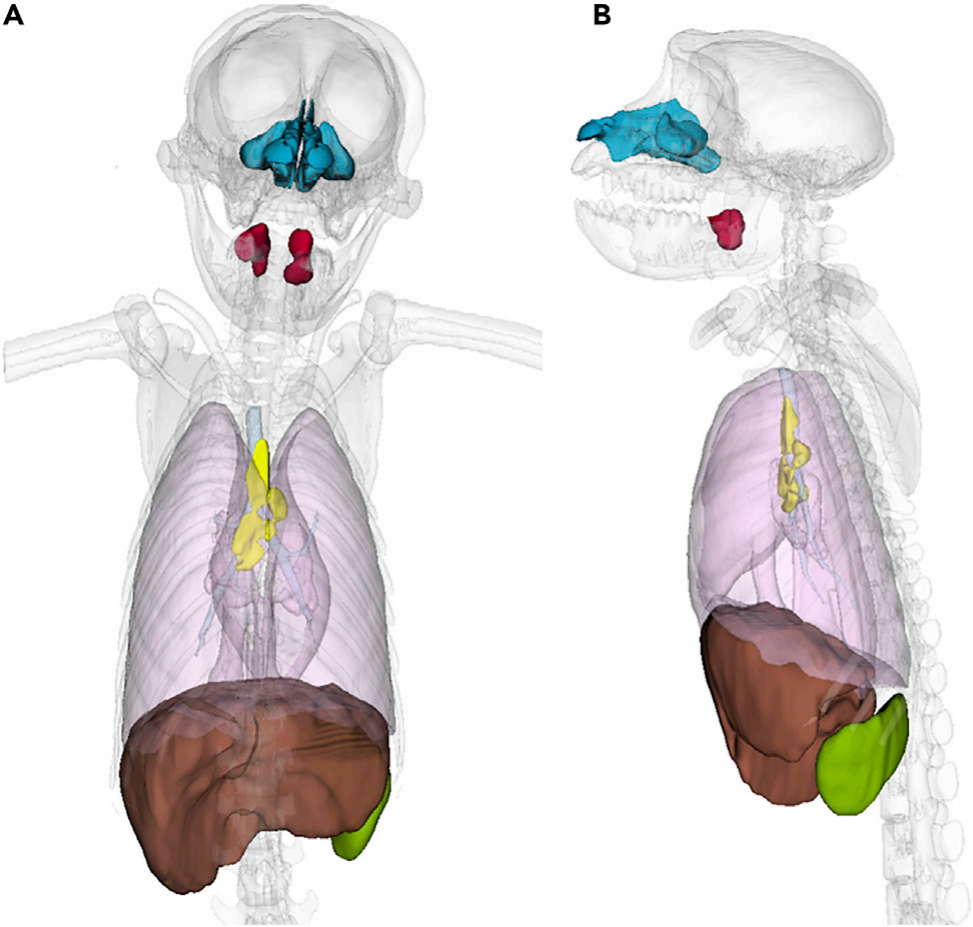
3D representation of regions of interest studied in SARS-CoV-2-infected macaques for [^18^F]-FDG uptake assessment (A) Coronal view, (B) Sagittal view. ROI of lungs (pink), liver (brown), spleen (green), nasal cavity (blue), tonsils (red), and lung-draining lymph nodes (yellow) are represented in 3D.

In addition to CT, 12 CM were followed by [^18^F]-FDG PET-CT. The [^18^F]-FDG accumulates *in vivo* in cells with highly metabolic activity, such as proliferating or activated immune cells. The main objective was to assess hypermetabolism linked to inflammation and cell infiltration occurring in the respiratory tract (lungs and nasal cavity), secondary lymphoid tissues (draining lymph nodes, spleen, and tonsils), and certain organs with high ACE2 expression, such as the liver and brain (Melin et al., 2020; Salamanna et al., 2020). These segmented regions of interest (ROIs) are illustrated in Figure 4.

**Figure 5:**
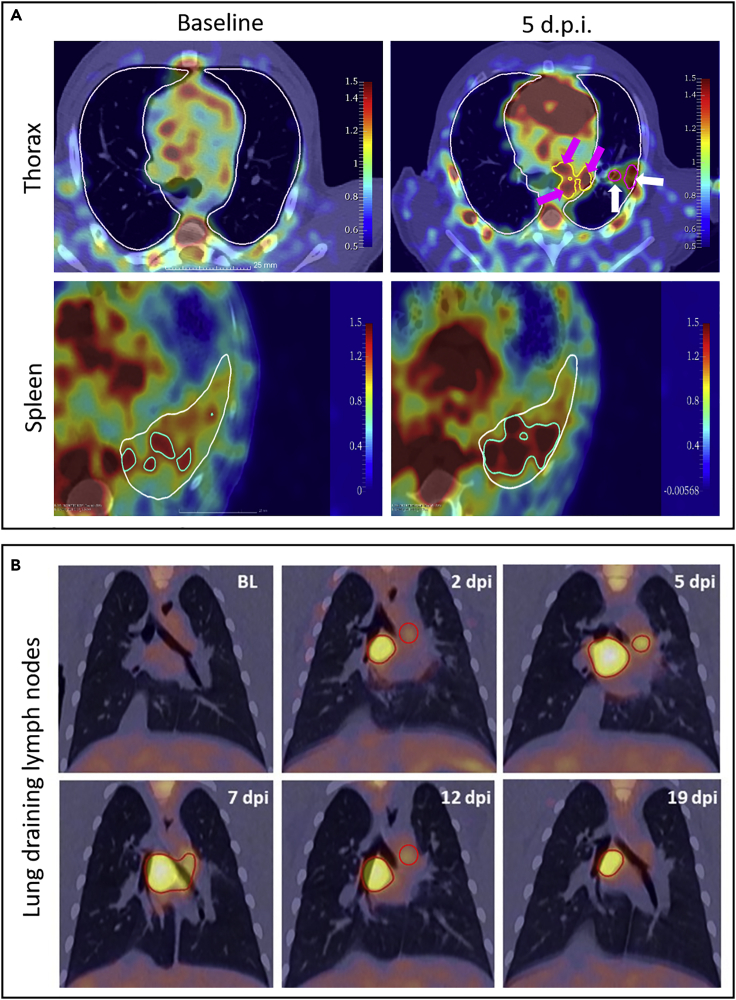
Representative PET/CT fusion images of [^18^F]-FDG signals in the thoracic area and spleen following infection in cynomolgus macaques (A) PET signal observed in the thoracic area before and at 5 days post-infection (d.p.i.), when an increased signal could be observed in lung-draining lymph nodes (pink arrows) and lung lesions (white arrows). Lungs were segmented (white delimitations) using CT. [^18^F]-FDG signal on PET-CT fused images observed in CT-segmented spleen (white delimitation) before and after SARS-CoV-2 exposure. Highest standardized uptake value (SUV) values in the spleen were delimitated in green to help in the comparison. (B) Frontal chest PET/CT fusion slices at baseline, 2 days post-infection (d.p.i.), 5 days.p.i., 7 days.p.i, 12 days.p.i, and 19 days.p.i. indicating longitudinal thoracic lymph node [^18^F]-FDG uptake over time (red circles indicate lymphatic [^18^F]-FDG signals).

We detected increased [^18^F]-FDG uptake in lung lesions (Figure 5A, white arrows) and lung-draining lymph nodes (Figure 5A, pink arrows). The increase of the [^18^F]-FDG signal could also be observed in the spleens of infected animals (Figure 5A). Longitudinal imaging over the first three weeks of infection in two CM also allowed assessment of the kinetics of lung-draining lymph node activation. We observed an increase in the [^18^F]-FDG signal in the lymph nodes over time during the first week of infection, followed by a progressive decrease over the following weeks (Figure 5B). Interestingly, signals in lung-draining LN persist much longer (3 weeks) than in the respiratory tract and in a time where virus could not be detected anymore in the tissues. We could then speculate that the [^18^F]-FDG signal in lung-draining lymph nodes may be mainly driven by the cellular activation of lymphocytes there.

**Figure 6:**
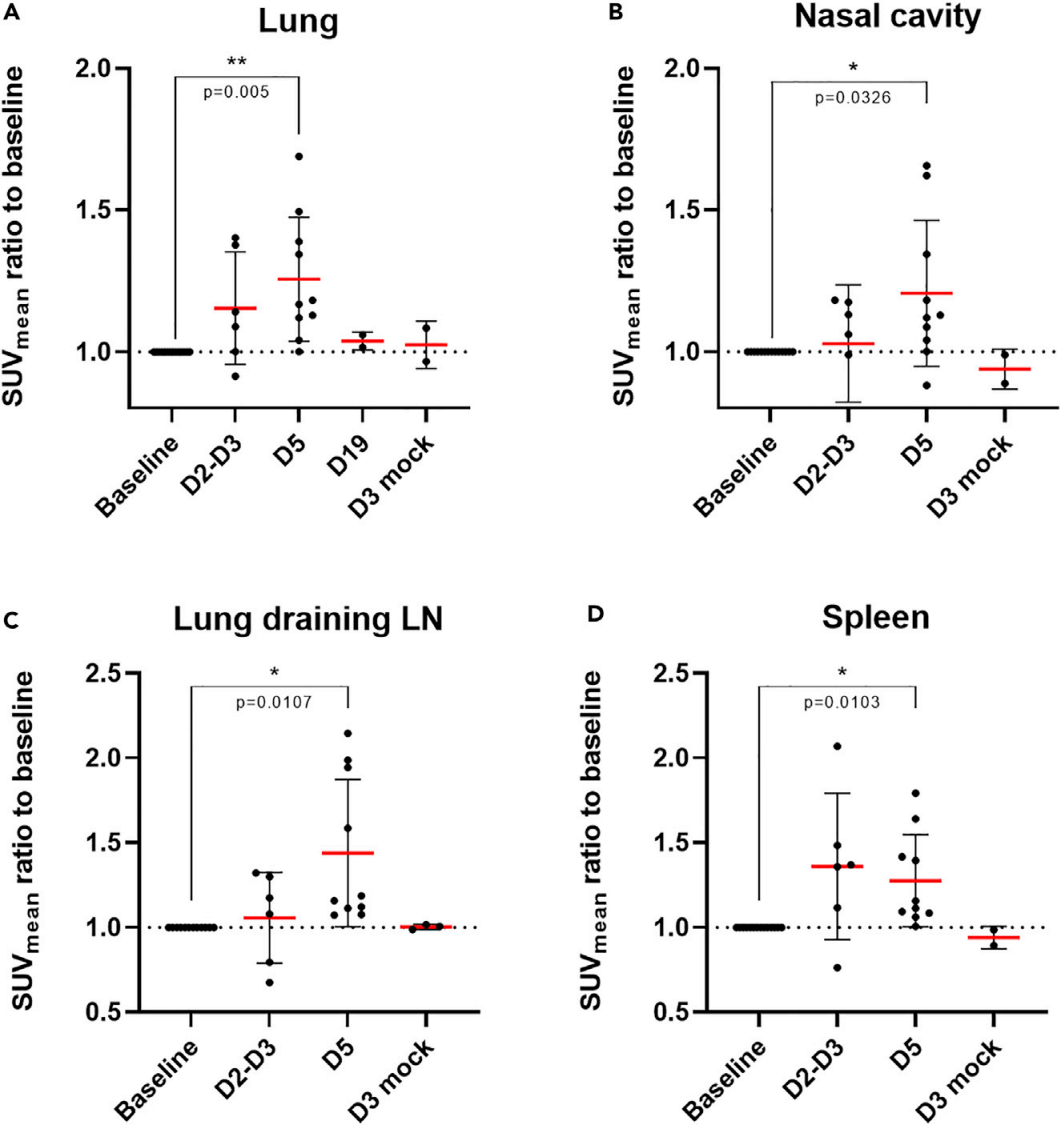
Quantitative [^18^F]-FDG-PET analysis in the lungs, nasal cavity, lung-draining lymph nodes, and spleen of SARS-CoV-2- or PBS-exposed cynomolgus macaques (A–D) Mean standard uptake values (SUV_mean_) are expressed as an individual ratio to baseline values in whole lungs (A), nasal cavity (B), lung-draining lymph nodes (C), and spleen (D) over time. ∗: p < 0.05, Paired t-tests. Mean values are represented in red with associated SD.

After organ segmentation and FDG uptake quantification, we detected a significant increase (p = 0.005) in the mean FDG uptake ratio (1.26 ± 0.22, expressed as a ratio to the baseline level) and the maximum FDG uptake ratio (1.19 ± 0.19) in the whole lungs by 5 days.p.i., with the SUVmax ratio even reaching significance earlier at 2–3 days.p.i. (1.07 ± 0.04) (Figures 6A and S5A). Interestingly, animals with the highest lung metabolic activity (ratios of 1.50 and 1.46) also had the highest CT scores (6 and 4, respectively), reflecting a correlation between lung lesions and induced metabolic activity. [^18^F]-FDG mean uptake also significantly increased (p = 0.03) at 5 days.p.i (1.22 ± 0.26, Figure 6B). in the nasal cavity of infected animals in comparison to baseline levels suggesting local inflammation or cellular activation in the nasal-associated lymphatic tissue. The same trend was observed in thoracic lung-draining lymph nodes with a significant increase (p = 0.01) of the mean FDG uptake observed by 5 days.p.i. compared to pre-infection levels (Figure 6C). FDG uptake by non-thoracic lymphoid organs was also affected very early after viral exposure. The mean metabolic activity of the spleen at 5 days.p.i. was significantly higher than at baseline levels (1.28 ± 0.27, p = 0.01 Figure 6D), with tonsils showing a similar pattern of uptake (1.25 ± 0.45, Figure S5B). No major increase of FDG uptake was detected in all these regions of interest in mock-infected animals (Figure 6).

Other organs of interest, such as the brain (1.09 ± 0.24) and liver (1.11 ± 0.24), did not show significant changes in metabolic activity detectable by PET at early time points (5 days.p.i.) following exposure (Figures S5C and S5D).

Similar [^18^F]-FDG uptake was observed for three RM, with no statistical differences found for [^18^F]-FDG uptake by the lungs or lung-draining lymph nodes relative to that by CM (Figure S6).

### Comparison of imaging data and virological or biological parameters

We first evaluated potential correlations between imaging data and viral RNA copy numbers in the various regions of interest of the airways. We compared the lung CT scores and contemporaneous viral genomic and subgenomic viral RNA levels for the nasopharyngeal cavity (genomic n = 45 and subgenomic n = 27) and bronchoalveolar lavage (BAL) (n = 26 and n = 21) of CM 2 to 5 days.p.i (Figure S7). No linear correlation could be found between either the nasopharyngeal (Figures S7A and S7C) or BAL (Figures S7B and S7D) viral load and the lung CT score. We also compared local [^18^F]-FDG uptake and the simultaneous genomic and subgenomic viral load in the nasopharyngeal cavity (genomic n = 9 and subgenomic n = 10) or in lung BAL (n = 6) of CM 2 to 5 days.p.i (Figure S8). No linear correlation could be found between either the nasopharyngeal (Figures S8A and S8B) or BAL (Figures S8C and S8D) viral load and associated local [^18^F]-FDG uptake.

As SARS-CoV-2 infection in CM induces an early significant decrease of circulating lymphocytes we also aimed at comparing this early lymphocyte decrease at 2 days.p.i. and the associated increase of lung-draining lymph nodes [^18^F]-FDG uptake. No linear correlation could be significantly found between those parameters (r = 0.43, p = 0.21), even if a negative trend could be observed (Figure S9).

### Use of computed tomography and positron emission tomography imaging for the assessment of treatment or vaccine efficacy in cynomolgus macaques

We previously performed CT on hydroxychloroquine (HCQ) or monoclonal antibody (COVA 1–18) treated animals or AAVCOVID-vaccinated macaques (Maisonnasse et al., 2020, 2021; Zabaleta et al., 2021). We compared their CT scores to those of the large cohort of control animals we described above (Table 2).

**Figure 7:**
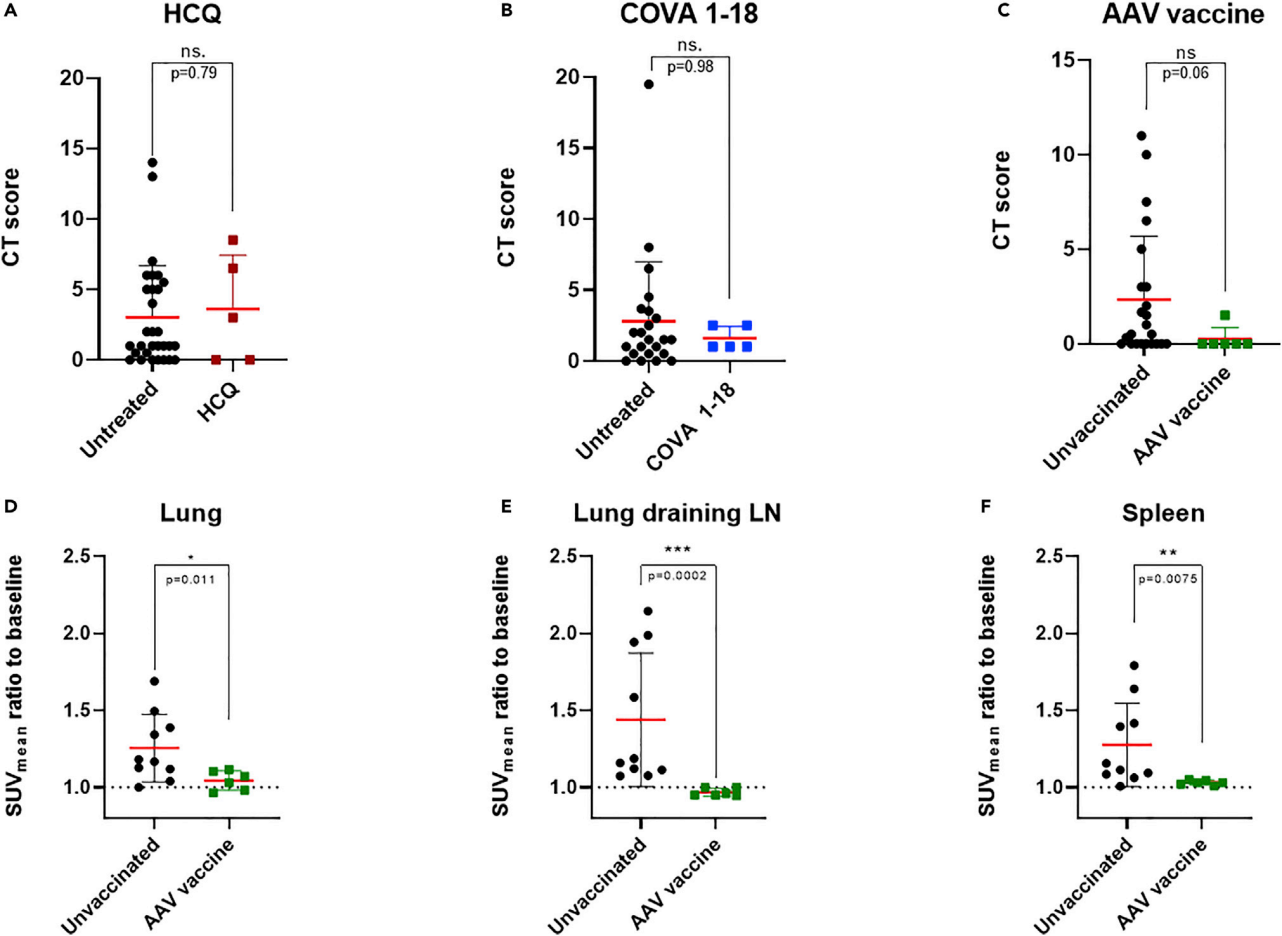
Comparison of treated or vaccinated cynomolgus macaques with control animals for CT scores and [^18^F]-FDG uptake by PET (A) CT scores at 2 days post-infection (d.p.i.) following exposure of macaques under hydroxychloroquine (HCQ, in maroon) or no treatment. (B) CT scores at 3 days.p.i. following exposure of macaques to a monoclonal COVA 1–18 treatment (blue) or not. (C) CT scores at 5 days.p.i. following exposure of macaques immunized with an AAV-based COVID-19 vaccine (green) or not. (D–F) Comparison of the mean standardized uptake value (SUV_mean_) ratios with the baseline for the lungs (D), lung-draining lymph nodes (E), and spleen (F) 5 days.p.i. of AAV-based COVID-19 vaccinated animals or unvaccinated animals. ns: non-statistically significant, ∗∗∗: p < 0.001, ∗∗: p < 0.01, ∗: p < 0.05. Mann–Whitney t-tests. Mean values are represented in red with associated SD.

There were no differences in the CT scores between HCQ-treated and control animals (p = 0.88, Figure 7A), in accordance with the absence of treatment efficacy in NHPs (Maisonnasse et al., 2020). On the contrary, there was a trend toward a decrease in the lung CT scores for COVA 1–18 treated animals relative to controls, with no score >5, but without reaching statistical significance, consistent with the proven antiviral property of this treatment (Maisonnasse et al., 2021). The same was true for AAVCOVID-vaccinated animals, which showed a lower lung CT score than non-vaccinated animals (p = 0.06, Figure 7C).

[^18^F]-FDG PET-CT imaging appeared to be better at detecting the effect of vaccines. In addition to CT scoring, AAVCOVID-vaccinated animals challenged with SARS-CoV-2 were followed by PET to assess [^18^F]-FDG uptake in the lungs, lung-draining lymph nodes, and spleen and were compared to non-vaccinated infected animals from this large cohort. All metabolic activities in these key organs following SARS-CoV-2 exposure of vaccinated animals showed a significant decrease (p = 0.01, p = 0.0002 and p = 0.008, respectively) in hypermetabolism (Figures 7D–7F) relative to non-vaccinated controls at 5 days.p.i.. These results obtained in the early days following SARS-CoV-2 infection indicate that the vaccinated animals were less reactive to viral challenge as a result of the near-sterilizing protection from the infection conferred by the AAVCOVID vaccine (Zabaleta et al., 2021).

## Discussion

The current COVID-19 pandemic has emphasized the crucial role of preclinical models in understanding the pathophysiology of human infection. Among several animal models of SARS-CoV-2 infection, NHP models thus far provide the closest pathology relative to humans, despite the absence of available models of severe cases. One key readout to monitor COVID-19 in patients is lung imaging, using either chest X-rays or CT. Such monitoring has been used in many NHP studies focusing on COVID-19 pathophysiology, treatments, and vaccines but have often been qualitative and not performed on any large cohorts or for extensive comparisons. Here, we followed 76 macaques, including 73 infected with SARS-CoV-2, by CT or [^18^F]-FDG PET/CT imaging, in addition to conventional biological parameters (viral load, cell blood counts, and plasma cytokine/chemokine concentrations), for readouts of COVID-19 pathophysiology. To our knowledge, this is the largest cohort study performed thus far.

As already described, we showed in this large cohort that SARS-CoV-2 infection-induced biological modifications very early after the infection. Infected macaques harbored viral RNA copies in the upper and lower respiratory tract with a peak of RNA copies at 2 days.p.i. in the nasopharynx followed by a progressive decrease within the next two weeks. In addition, a transient decrease of circulating leukocytes (mainly driven by a lymphocyte count drop) was detected as soon as 2 days.p.i. Some pro-inflammatory cytokines/chemokines such as IL-1RA, MCP-1, and IL-15 also increased transiently at the very early days following SARS-CoV-2 infection.

Regarding imaging follow-up, we were able to detect and quantify at the very early days following infection all typical radiological features of COVID-19 in the lungs of macaques by CT, with no statistical differences in scores between cynomolgus or rhesus species, nor the initial exposure dose. We were also able to reproduce the heterogeneity of the lung burden that can be observed – or, on the contrary, not observed - in humans. Indeed, most infected people remain asymptomatic or with moderate symptoms that do not require chest imaging. Thus, these data consolidate the accuracy of cynomolgus and rhesus macaque models for COVID-19 studies, as already described for smaller cohorts (Salguero et al., 2021). However, as also shown here, there is no severe NHP model of SARS-CoV-2 infection available at a large scale. Indeed, the monkeys used worldwide, as in this study, are generally young adults with no major comorbidities. We also showed that semi-quantitative CT scoring correlates well with quantitative PCLH analysis, thus addressing the potential bias due to operator-dependent CT scoring for the quantification of lung lesions. This may validate CT scoring, as it would be faster and easier for radiologists to use. Conversely, we found no correlation between CT lung scoring and simultaneous estimated SARS-CoV-2 genomic or subgenomic RNA titers, either in the nasopharynx or the lungs. This observation suggests that both imaging and RT-qPCR techniques must be carried out to properly assess and monitor the evolution of the pathology. This may be particularly true for patients, for whom false-negative PCRs are more frequent due to more heterogeneous sampling. The lung burden due to SARS-CoV-2 infection thus appears to be more highly associated with complex immune responses and is not strictly virus-dependent. We addressed this issue by showing that, in addition to CT, [^18^F]-FDG PET imaging can be used for the quantitative monitoring of local metabolic activation (linked to inflammation) throughout the course of the disease following exposure to SARS-CoV-2 and especially during the acute phase of the infection. Thus, we quantified a significant increase of local inflammation occurring in lungs, as well as in the nasal cavity during the course of the disease. We additionally monitored a significant increase of cellular activation in lung-draining lymph nodes, the spleen, and tonsils from the first days following exposure compared to uninfected baseline inflammatory levels, without any sequential surgical or necropsy interventions. This additional minimally invasive functional readout, also used in the clinic for other diagnostic purposes, such as for cancer, could thus be implemented in the future to monitor disease progression in hospitalized patients with COVID-19. Moreover, as for CT scoring, the observed local inflammation did not appear to correlate with associated local viral shedding. Thus, this inflammation-targeted imaging approach provides an additional monitoring tool for COVID-19 in various organs in NHPs and also improves our understanding of COVID-19 pathophysiology as it was already conducted previously in other macaque models of infectious disease such as SIV (Scharko et al., 1996). However, as [^18^F]-FDG only points out cellular activation without discriminating the phenotype of the cells, more in-depth analysis must be carried out to investigate what kind of activated cells contribute to the lung burden or disease progression. These imaging readouts of SARS-CoV-2 infection in NHPs were then compared to some of our imaging data from previous treatment or vaccine efficacy tests. We were able to show that lung CT scoring could be a fast readout of vaccine or treatment efficacy regarding severe lesions or average scoring but the heterogeneity of lung CT scores may prevent from reaching statistical significance in small cohorts. On the contrary, we showed in this study that early PET follow-up of [^18^F]-FDG uptake of key organs of interest such as lungs, lung-draining lymph nodes, or spleen could be a significant readout of vaccine efficacy. These imaging and molecular readouts produced using large NHP cohorts, together with other biological readouts, such as serum antibody neutralization titers, could also be useful in the near future to build automatic, deep-learning based, algorithms that may predict disease outcomes in animals, as already reported for patients (Chassagnon et al., 2021). Overall, these large NHP cohort data suggest that CT, eventually combined with [^18^F]-FDG PET imaging, for the study of SARS-CoV-2 infected, NHPs can produce strong and transposable readouts to monitor COVID-19 disease, as well as the efficacy of future treatments and vaccine candidates.

### Limitations of the study

We provide imaging data on SARS-CoV-2 exposed animals with the initial “Wuhan” strain. The first limitation may then reside in the different imaging patterns that can be induced with other variants such as Delta or Omicron and this requires further studies. The second limitation is that most of our animals were young and do not show extended lung lesions and acute lung inflammation on contrary to hospitalized patients.

## STAR★Methods

### Key resources table

**Table:** 

REAGENT or RESOURCE	SOURCE	IDENTIFIER
**Bacterial and virus strains**		

SARS-CoV-2 (hCoV-19/France/IDF0372/2020 strain)	Institut Pasteur	EPI_ISL_410720 (GISAID ID)

**Critical commercial assays**		

MILLIPLEX MAP Non-Human Primate Cytokine Magnetic Bead Panel - Immunology Multiplex Assay	Millipore	PRCYTOMAG-40K
Cytokine/Chemokine/Growth Factor 37-Plex NHP ProcartaPlex™ Panel	ThermoFischer	EPX370-40045-901

**Experimental models: Organisms/strains**		

Cynomolgus macaques	Noveprim	N/A
Rhesus macaques	Station de Primatologie du CNRS	N/A
Rhesus macaques	Silabe	N/A
Rhesus macaques	Hartelust	N/A

**Oligonucleotides**		

RdRp-IP4 primer, forward – GGTAACTG GTATGATTTCG	https://www.who.int/docs/default-source/coronaviruse/real-time-rt-pcr-assays-for-the-detection-of-sars-cov-2-institut-pasteur-paris.pdf?sfvrsn=3662fcb6_2	N/A
RdRp-IP4 primer, reverse - CTGGTCAAG GTTAATATAGG	https://www.who.int/docs/default-source/coronaviruse/real-time-rt-pcr-assays-for-the-detection-of-sars-cov-2-institut-pasteur-paris.pdf?sfvrsn=3662fcb6_2	N/A
RdRp-IP4 primer probe P - TCATACAAA CCACGCCAG G	https://www.who.int/docs/default-source/coronaviruse/real-time-rt-pcr-assays-for-the-detection-of-sars-cov-2-institut-pasteur-paris.pdf?sfvrsn=3662fcb6_2	N/A
sgLead SARSCoV2-forward - CGATCTCTT GTAGATCTGTTCTC	Corman et al., 2020	N/A
E-Sarbeco-reverse primer - ATATTGCA GCAGTACGCACACA	Corman et al., 2020	N/A
E-Sarbeco probe HEXACACTAGCCATC CTTACTGCGCTTCG-BHQ1	Corman et al., 2020	N/A

**Software and algorithms**		

GraphPad Prism v8	GraphPad	N/A
INTELLISPACE PORTAL 8 software	Philips Healthcare	https://www.philips.fr/healthcare/product/HC881062/intellispace-portal-80-all-your-advanced-analysis-needs-one-comprehensive-solution
3D slicer v4.11	Slicer (Open source)	https://www.slicer.org/

**Other**		

HMX A/L analyser	Beckman Coulter	N/A
Philips Vereos® PET/CT	Philipas Healthcare	N/A

### Resource availability

#### Lead contact

Further information and requests for resources and reagents should be directed to and will be fulfilled by the Lead Contact, Thibaut Naninck (thibaut.naninck@cea.fr).

#### Material availability

This study did not generate new unique reagents.

### Experimental model and subject details

#### Virus

SARS-CoV-2 virus (hCoV-19/France/lDF0372/2020 strain) was isolated by the National Reference Center for Respiratory Viruses (Institut Pasteur, Paris, France) and produced by two passages on Vero E6 cells in DMEM (Dulbecco’s Modified Eagles Medium) without FBS, supplemented with 1% P/S (penicillin at 10,000 U mL^−1^ and streptomycin at 10,000 mg mL^−1^) and 1 mg mL^−1^ TPCK-trypsin at 37°C in a humidified CO_2_ incubator and titrated on Vero E6 cells.

#### Ethics and biosafety statement

Male and female cynomolgus macaques (*Macaca fascicularis*), originating from Mauritian AAALAC certified breeding centers, were used in this study. Rhesus macaques (*Macaca mulata*) were provided by European breeding centers (Station de Primatologie de Rousset sur Arc, SILABE and Hartelust). All animals were housed at the IDMIT infrastructure facilities (CEA, Fontenay-aux-roses) under BSL-2 and BSL-3 containment, when necessary (Animal facility authorization #D92-032-02, Préfecture des Hauts de Seine, France) and in compliance with European Directive 2010/63/EU, French regulations, and the Standards for Humane Care and Use of Laboratory Animals of the Office for Laboratory Animal Welfare (OLAW, assurance number #A5826-01, US). The protocols were approved by the institutional ethical committee “Comité d’Ethique en Expérimentation Animale du Commissariat à l’Energie Atomique et aux Energies Alternatives” (CEtEA #44) under statement number A20-011. The study was authorized by the “Research, Innovation and Education Ministry” under registration number APAFIS#24434-2020030216532863.

#### Animal and study design

Sixty-one cynomolgus macaques and 15 rhesus macaques, all young adults, were randomly assigned between dose groups as described in Table S1. All animals were then exposed to a total dose of 10^5^, 10^6^, or 10^7^ PFU of SARS-CoV-2 (BetaCoV/France/IDF/0372/2020, passaged twice in VeroE6 cells) via a combination of the intranasal and the intra-tracheal routes (day 0), using atropine (0.04 mg kg^−1^) for pre-medication and ketamine (5 mg kg^−1^) with medetomidine (0.05 mg kg^−1^) for anesthesia. Among them, three cynomolgus macaques were mock exposed using saline with the same protocol. Animals were observed daily, and clinical exams were performed at baseline, daily for one week, and then twice weekly on anesthetized animals using ketamine (5 mg kg^−1^) and medetomidine (0.05 mg kg^−1^). Nasopharyngeal specimens were collected with swabs (Viral Transport Medium, CDC, DSR-052-01). Broncho-alveolar lavages (BAL) were performed using 50 mL sterile saline. All specimens were stored between 2°C and 8°C until analysis by RT-qPCR with a plasmid standard concentration range containing an RdRp gene fragment, including the RdRp-IP4 RT-PCR target sequence. SARS-CoV-2 E gene subgenomic mRNA (sgRNA) levels were assessed by RT-qPCR using previously described primers and probes (Corman et al., 2020; Wölfel et al., 2020) :leader-specific primer sgLeadSARSCoV2-F CGATCTCTTGTAGATCTGTTCTC, E-Sarbeco-R primer ATATTGCAGCAGTACGCACACA and E-Sarbeco probe HEX-ACACTAGCCATCCTTACTGCGCTTCG-BHQ1. The protocol describing the procedure for the detection of SARS-CoV-2 is available on the WHO website (https://www.who.int/docs/default-source/coronaviruse/real-time-rt-pcr-assays-for-the-detection-of-sars-cov-2-institut-pasteur-paris.pdf?sfvrsn=3662fcb6_2). Blood cell counts were determined from EDTA-treated blood samples using a HMX A/L analyser (Beckman Coulter). Cytokines were quantified in EDTA-treated plasma using NHP Procarta (ThermoFisher Scientific) or NHP Milliplex immunoassays (Millipore) for IL-1RA, MCP-1 (also known as CCL-2), IL-15, IFNγ, IFNα, IL-6 and a Bioplex 200 analyser (Bio-Rad) according to manufacturer’s instructions. Chest CT or PET-CT were performed at least at baseline and at early time points (2 d.p.i to 5 d.p.i.) on anesthetized animals.

### Method details

#### CT and PET-CT acquisition

All imaging acquisition was performed using the Digital Photon Counting (DPC) PET-CT system (Vereos-Ingenuity, Philips) implemented in the BSL-3 laboratory.

For PET acquisitions, animals were fasted for at least 8 h before the imaging session. These sessions were always performed in the same experimental conditions (acquisition time and animal order) to limit [^18^F]-FDG-PET experimental bias. Animals were first anesthetized with ketamine (10 mg kg^−1^) + medetomidine (0.05 mg kg^−1^), intubated, and then maintained under 2% isoflurane and placed in a supine position on a warming blanket (Bear Hugger, 3M) on the machine bed with monitoring of the cardiac rate, oxygen saturation, and temperature.

For PET-CT acquisitions, CT was performed twice, once under breath-hold (for CT anatomical segmentation) and again 5 min prior to PET acquisition for attenuation correction and anatomical localization.

The CT detector collimation used was 64 × 0.6 mm, the tube voltage was 120 kV, and the intensity was approximately 150 mAs. The intensity was regulated by automatic dose optimization tools (Dose Right, Z-DOM, 3D-DOM by Philips Healthcare). Chest-CT images were reconstructed with a slice thickness of 1.25 mm and an interval of 0.63 mm. Whole-body CT images were reconstructed with a slice thickness of 1.5 mm and an interval of 0.75 mm.

A whole-body PET scan (4–5 bed positions, 3 min/bed position) was performed approximately 45 min post-injection of 4.2 ± 1.0 MBq kg^−1^ of [^18^F]-FDG via the saphenous vein. PET images were reconstructed onto a 256 × 256 matrix using OSEM (3 iterations, 15 subsets).

The same CT parameters were used for CT-scan sessions only. A second CT scan was performed on a small portion of the animals (n = 6) to better visualize the lymph nodes in the chest area. Iodine contrast agent (Vizipaque 320 mg I mL^−1^, GE Healthcare, 3 mL kg^−1^) was automatically injected (Medrad CT Stellant® injector, Bayer) as a bolus in the saphenous vein 20 s prior to starting CT acquisition.

#### Image analysis

PET and CT images were analyzed using INTELLISPACE PORTAL 8 (Philips Healthcare) and 3DSlicer (open-source tool).

All CT lung images had the same window level of −300 and a window width of 1,600.

Pulmonary lesions were defined as ground-glass opacity, crazy-paving pattern, or consolidations, as previously described (Brouwer et al., 2021; Maisonnasse et al., 2020, 2021; Pan et al., 2020; Shi et al., 2020). Two to three individuals assessed the lesion features detected by CT imaging independently and the final CT score results (maximum score of 84) were determined by consensus. Pre-existing background lesions or lesions induced by experimental atelectasis were scored 0.

For segmentation, various regions of interest (lung, lung-draining lymph nodes, nasal cavities, spleen, liver, brain, tonsils) were semi-automatically contoured according to anatomical information. A 3D volume of interest (VOI) was interpolated from several ROIs in different image slices to cover the entire organ. Considering the partial volume, [^18^F]-FDG accumulation in the VOIs was given as a standardized uptake value (SUVmean, SUVmax) and organ-to-background ratios (or ratio to baseline) were calculated for each animal.

Using the same anatomical lung segmentation previously described, we performed quantitative CT analysis for the high-density lung lesions. A new ROI was defined choosing a Hounsfield value between −300 and 150. Finally, the percentage of the change in volume from the baseline (PCLH) was calculated for each timepoint of interest.

### Quantification and statistical analysis

Statistical analyses were carried out using linear regression and paired or Mann-Whitney unpaired t-tests (Significance p < 0.05) in GraphPad Prism software (v8.3.0).

## Data Availability

Data: The raw data supporting the findings of the study are available from the lead contact upon request. Code: This paper does not report original code General statement: Any additional information required to reanalyze the data reported in this paper is available from the lead contact upon request.
