# Supplementary material for: Computed tomography and [18F]-FDG PET imaging provide additional readouts for COVID-19 pathogenesis and therapies evaluation in non-human primates
Source: iScience. 2022 Mar 17;25(4):104101. doi: 10.1016/j.isci.2022.104101 (PMC8926429; doi:10.1016/j.isci.2022.104101)
Supplement: Document S1. Figures S1–S9, Tables S1, and S2 [file mmc1.pdf]

iScience, Volume *25*

Supplemental information

Computed tomography and [^{18}F]-FDG PET imaging provide additional readouts for COVID-19 pathogenesis and therapies evaluation in non-human primates

Thibaut Naninck, Nidhal Kahlaoui, Julien Lemaitre, Pauline Maisonnasse, Antoine De Mori, Quentin Pascal, Vanessa Contreras, Romain Marlin, Francis Relouzat, Benoît Delache, Cécile Hérate, Yoann Aldon, Marit van Gils, Nerea Zabaleta, Raphaël Ho Tsong Fang, Nathalie Bosquet, Rogier W. Sanders, Luk H. Vandenberghe, Catherine Chapon, and Roger Le Grand

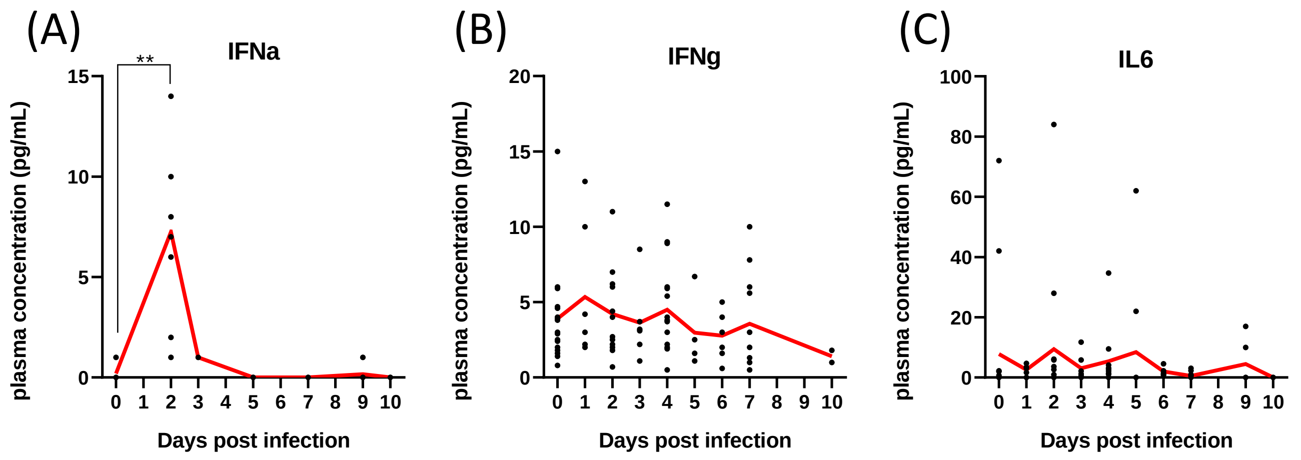

Figure S1, related to Figure 1. Plasma concentrations of IFNα (A), IFNγ (B) and IL-6 (C) over time in SARS-CoV-2 infected cynomolgus macaques. Paired t-tests: **: $p < 0.01$

Code	Species	Sex	Birth date	Weight at inclusion	Exposure dose (PFU)	Imaging modality	Early time point	W1 time point	W2 time point	W3 time point
MF1	Macaca fascicularis	F	04/03/2017	4,14	10^7	PET-CT	yes	yes	yes	yes
MF2	Macaca fascicularis	F	11/03/2017	2,69	10^7	PET-CT	yes	yes	yes	yes
MF3	Macaca fascicularis	F	06/03/2017	3,2	10^6	PET-CT	yes	no	no	no
MF4	Macaca fascicularis	F	11/03/2017	3,53	10^6	PET-CT	yes	no	no	no
MF5	Macaca fascicularis	M	02/03/2017	2,83	10^6	PET-CT	yes	no	no	no
MF6	Macaca fascicularis	F	26/11/2017	2,64	10^6	PET-CT	yes	no	no	no
MF7	Macaca fascicularis	M	22/02/2017	3,66	10^6	CT	yes	no	yes	no
MF8	Macaca fascicularis	F	15/12/2016	2,79	10^6	CT	yes	no	yes	no
MF9	Macaca fascicularis	F	20/12/2016	3,24	10^6	CT	yes	no	yes	no
MF10	Macaca fascicularis	F	21/12/2016	3,45	10^6	CT	yes	no	yes	no
MF11	Macaca fascicularis	F	04/01/2017	3,47	10^6	CT	yes	no	yes	no
MF12	Macaca fascicularis	M	14/02/2017	4,15	10^6	CT	yes	no	yes	no
MF13	Macaca fascicularis	M	02/03/2017	3,11	10^6	CT	yes	no	yes	no
MF14	Macaca fascicularis	M	04/03/2017	3,41	10^6	CT	yes	no	yes	no
MF15	Macaca fascicularis	F	10/05/2013	4,7	10^6	CT	yes	no	no	no
MF16	Macaca fascicularis	F	03/02/2014	6,88	10^6	CT	yes	no	no	no
MF17	Macaca fascicularis	F	16/10/2013	6,31	10^6	CT	yes	no	no	no
MF18	Macaca fascicularis	F	11/11/2013	4,98	10^6	CT	yes	no	no	no
MF19	Macaca fascicularis	F	30/10/2013	3,82	10^6	CT	yes	no	no	no
MF20	Macaca fascicularis	F	02/03/2017	2,94	10^6	CT	yes	no	no	no
MF21	Macaca fascicularis	F	04/03/2017	4,21	10^6	CT	yes	no	no	no
MF22	Macaca fascicularis	F	04/04/2017	3,25	10^6	CT	yes	no	no	no
MF23	Macaca fascicularis	F	07/03/2017	3,77	10^6	CT	yes	no	no	no
MF24	Macaca fascicularis	F	29/10/2013	4,02	10^6	CT	yes	no	no	no
MF25	Macaca fascicularis	F	11/07/2015	5,16	10^6	CT	yes	yes	yes	no
MF26	Macaca fascicularis	F	24/06/2015	2,79	10^6	CT	yes	yes	yes	no
MF27	Macaca fascicularis	F	21/08/2015	4,03	10^6	CT	yes	yes	yes	no
MF28	Macaca fascicularis	F	18/07/2015	5,46	10^6	CT	yes	yes	yes	no
MF29	Macaca fascicularis	M	03/03/2017	5,53	10^6	CT	yes	yes	no	no
MF30	Macaca fascicularis	F	22/08/2017	3,14	10^6	CT	yes	yes	no	no
MF31	Macaca fascicularis	F	02/01/2016	3,05	10^6	CT	yes	yes	no	no
MF32	Macaca fascicularis	F	03/04/2017	3,16	10^6	CT	yes	yes	no	no
MF33	Macaca fascicularis	F	13/03/2017	3,63	10^6	CT	yes	yes	no	no
MF34	Macaca fascicularis	M	16/03/2017	6,19	10^6	CT	yes	yes	no	no
MF35	Macaca fascicularis	F	18/04/2017	2,71	10^5	PET-CT	yes	no	yes	no
MF36	Macaca fascicularis	F	20/03/2017	4,41	10^5	PET-CT	yes	no	yes	no
MF37	Macaca fascicularis	M	07/04/2017	3,75	10^5	PET-CT	yes	no	yes	no
MF38	Macaca fascicularis	F	24/03/2017	3,16	10^5	PET-CT	yes	no	yes	no
MF39	Macaca fascicularis	F	12/04/2017	3,71	10^5	PET-CT	yes	no	yes	no
MF40	Macaca fascicularis	M	06/04/2017	5,1	10^5	PET-CT	yes	no	yes	no
MF41	Macaca fascicularis	M	10/04/2017	4,98	10^5	CT	yes	yes	yes	no
MF42	Macaca fascicularis	M	12/04/2017	6,6	10^5	CT	yes	yes	yes	no
MF43	Macaca fascicularis	M	05/04/2017	4,58	10^5	CT	yes	yes	yes	no
MF44	Macaca fascicularis	M	04/04/2017	3,91	10^5	CT	yes	yes	yes	no
MF45	Macaca fascicularis	M	08/03/2017	4,92	10^5	CT	yes	no	yes	no
MF46	Macaca fascicularis	M	25/05/2017	4,56	10^5	CT	yes	no	yes	no
MF47	Macaca fascicularis	M	25/04/2017	3,61	10^5	CT	yes	no	no	no
MF48	Macaca fascicularis	M	18/04/2017	4,76	10^5	CT	yes	no	yes	no
MF49	Macaca fascicularis	M	04/04/2017	3,92	10^5	CT	yes	no	no	no
MF50	Macaca fascicularis	M	01/05/2017	5,36	10^5	CT	yes	no	no	no
MF51	Macaca fascicularis	M	12/05/2017	4,1	10^5	CT	yes	no	no	no
MF52	Macaca fascicularis	M	01/04/2017	4,28	10^5	CT	yes	no	yes	no
MF53	Macaca fascicularis	M	17/02/2017	3,97	10^6	CT	yes	yes	no	no
MF54	Macaca fascicularis	F	13/12/2016	3,46	10^6	CT	yes	yes	no	no
MF55	Macaca fascicularis	F	05/01/2017	3,19	10^6	CT	yes	yes	no	no
MF56	Macaca fascicularis	M	15/02/2017	4,08	10^6	CT	yes	yes	no	no
MF57	Macaca fascicularis	M	06/03/2017	4,58	10^6	CT	yes	yes	no	no
MF58	Macaca fascicularis	M	28/02/2017	4,76	10^6	CT	yes	yes	no	no
MF59	Macaca fascicularis	M	21/03/2017	6,35	0	PET-CT	yes	no	no	no
MF60	Macaca fascicularis	F	03/04/2017	4,27	0	PET-CT	yes	no	no	no
MF61	Macaca fascicularis	F	24/08/2017	3,11	0	PET-CT	yes	no	no	no
MM1	Macaca mulata	F	24/05/2015	5,65	10^7	PET-CT	yes	yes	yes	yes
MM2	Macaca mulata	F	17/04/2015	5,32	10^7	PET-CT	yes	yes	yes	yes
MM3	Macaca mulata	F	23/04/2015	5,39	10^6	PET-CT	yes	no	no	no
MM4	Macaca mulata	F	16/04/2015	6,31	10^5	CT	yes	no	yes	no
MM5	Macaca mulata	F	23/04/2015	6,57	10^5	CT	yes	no	yes	no
MM6	Macaca mulata	F	18/03/2015	6,28	10^5	CT	yes	no	yes	no
MM7	Macaca mulata	F	19/04/2015	5,76	10^5	CT	yes	no	yes	no
MM8	Macaca mulata	M	21/01/2003	8,84	10^5	CT	yes	yes	no	no
MM9	Macaca mulata	F	27/07/2003	8,34	10^5	CT	yes	yes	no	no
MM10	Macaca mulata	F	22/07/2011	8,04	10^5	CT	yes	yes	no	no
MM11	Macaca mulata	M	31/05/2014	9,83	10^5	CT	yes	yes	no	no
MM12	Macaca mulata	F	23/08/2009	5,28	10^5	CT	yes	yes	no	no
MM13	Macaca mulata	M	18/05/2008	7,83	10^5	CT	yes	yes	no	no
MM14	Macaca mulata	M	21/12/2015	5,47	10^5	CT	yes	yes	no	no
MM15	Macaca mulata	F	03/04/2014	6,62	10^5	CT	yes	yes	no	no

Table S1, related to Table 1. Individual animal data. Species, sex, birth date, weight, exposure dose, imaging modality used for monitoring, and the timing of imaging are presented. PFU: plaque-forming unit

Operator	Date	Time point	Animal	Localisation	GGO	Pleural thickenning	Crazy-paving pattern	Consolidation	Lesion extension	Pleural effusion	Total score
				Cranial right lobe							
				Middle right lobe							
				Caudal right lobe							
				Cranial left lobe culmen							
				Cranial left lobe lingula							
				Caudal left lobe							
				Accessory lobe							
				Pleural space							
				Total							

Lesion type	Score
None	0
GGO	1
Pleural thickenning	1
Crazy-paving pattern	3
Consolidation	3

Lesion extension	Score
None	0
< 25% of the lobe	1
25 < x < 50% of the lobe	2
50 < x < 75% of the lobe	3
75 < x < 100% of the lobe	4

Pleural effusion	Score
None	0
Slight	1
Mild	2
Severe	3

Table S2, related to figure 2-3. CT scoring grid and scales.

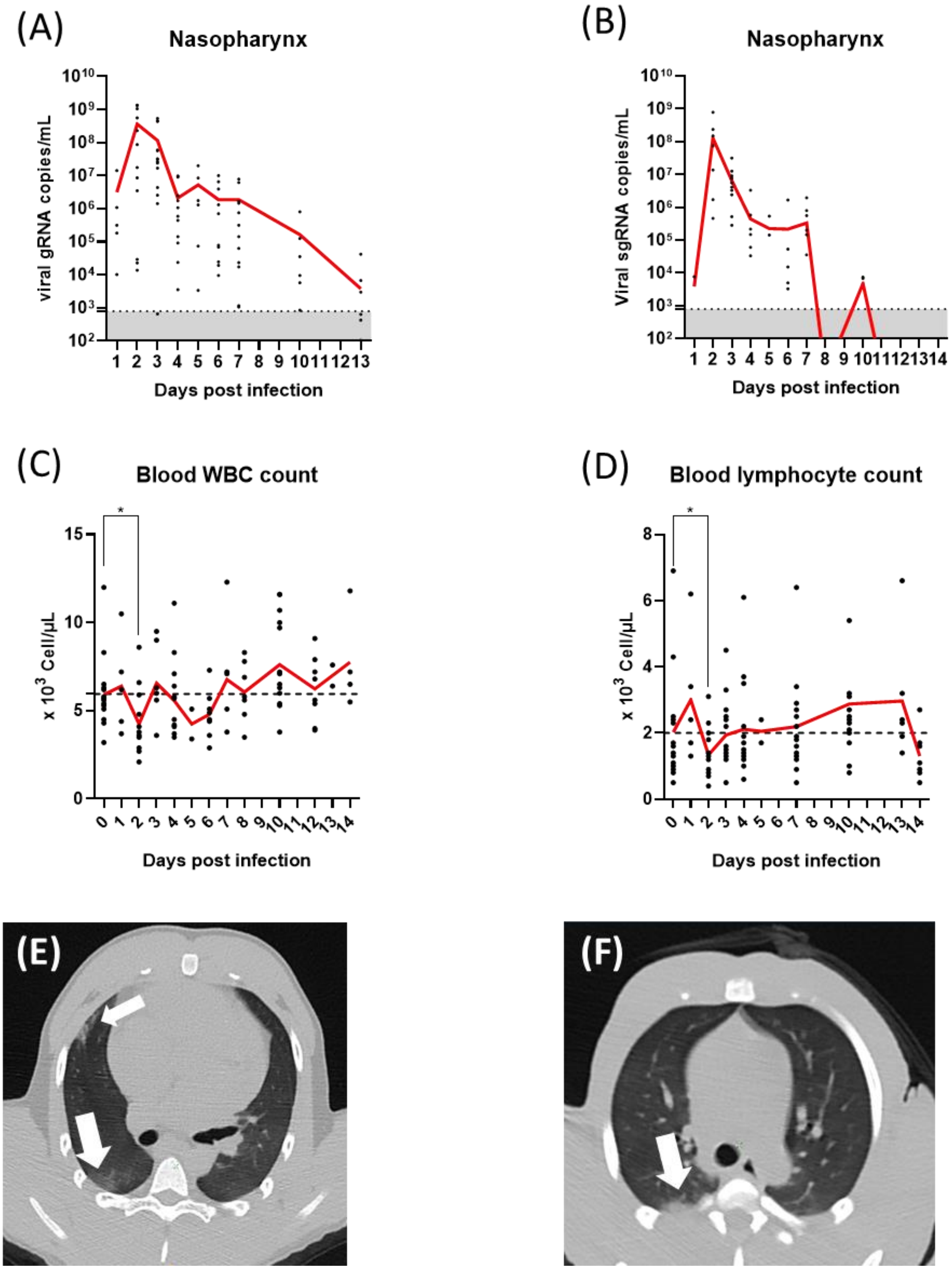

Figure S2 related to Figure 1-2. Monitoring of SARS-CoV-2 infection in Rhesus macaques. Nasopharynx genomic (A) and subgenomic (B) SARS-CoV-2 RNA copies per milliliter evolution over time following infection. Dotted lines represent the limit of quantification. White blood cells (C) and lymphocytes (D) counts in blood over time. (C-D) Dotted lines represent the average baseline values. (E-F) Chest CT images with low-density ground-glass opacity (GGOs) (E, arrows) and higher density pleural GGO (F, arrow).

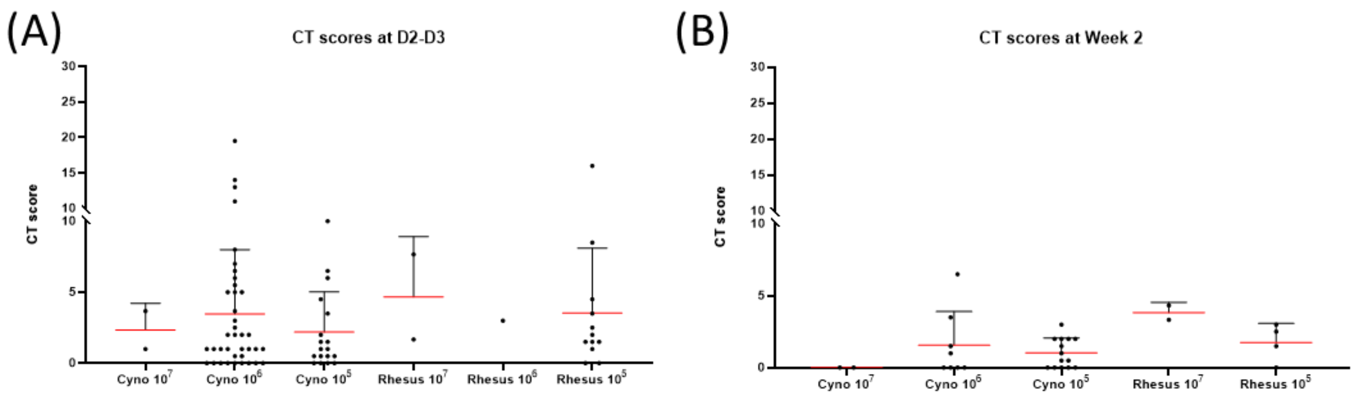

Figure S3, related to Figure 3. Chest CT scan scoring data. (A-B) CT scores evaluated at 2-3 days post-infection (d.p.i.) (A) or at the second week following infection (B) in SARS-CoV-2-exposed cynomolgus and rhesus macaques according to the inoculum dose in plaque-forming units (PFU).

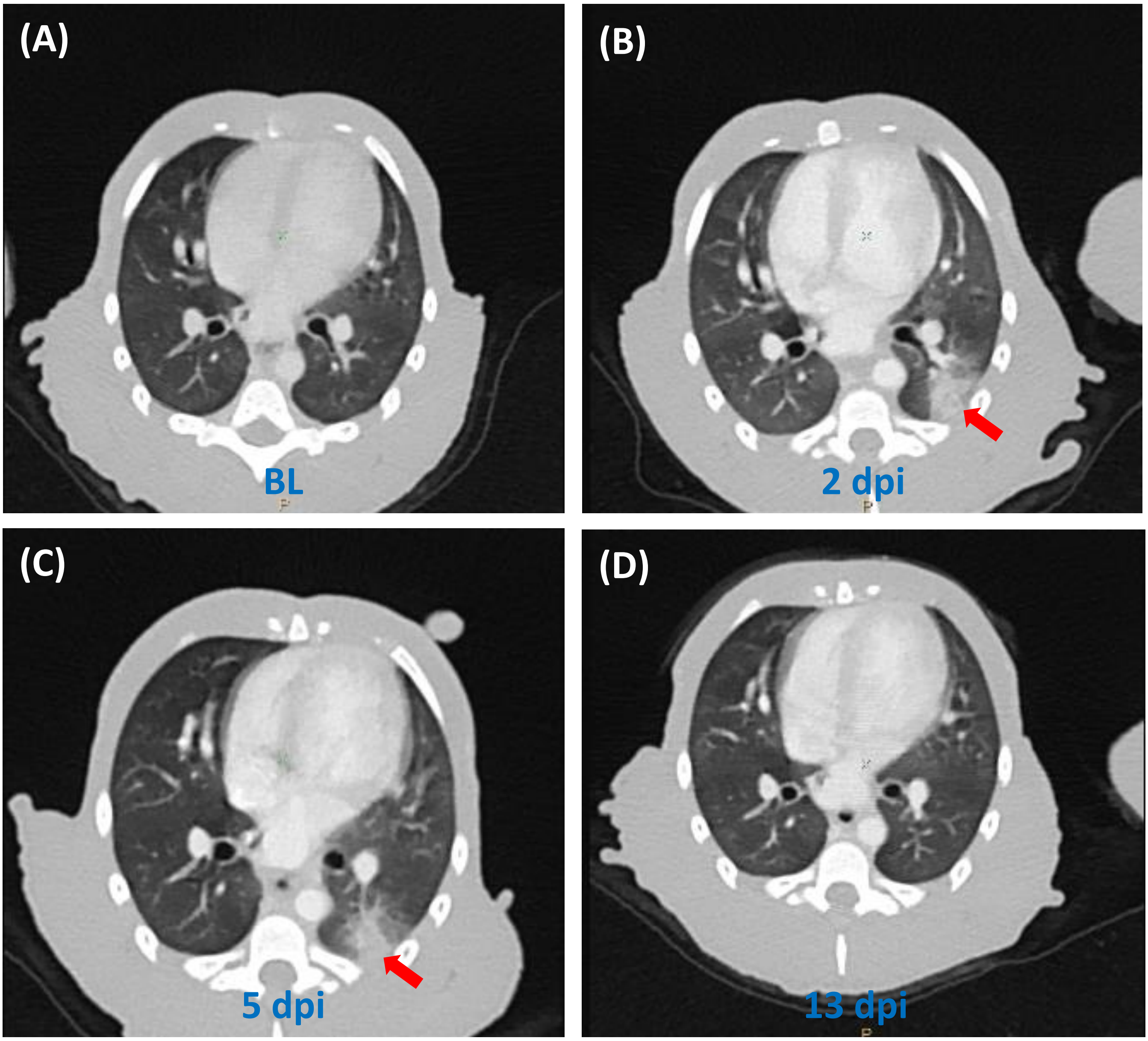

Figure S4, related to Figure 3. Example of the evolution of a SARS-CoV-2 lung lesion over time by CT in a cynomolgus macaque. Chest CT transversal slices performed at baseline (A), 2 days post-infection (d.p.i.) (B), 5 d.p.i. (C), and 13 d.p.i (D).

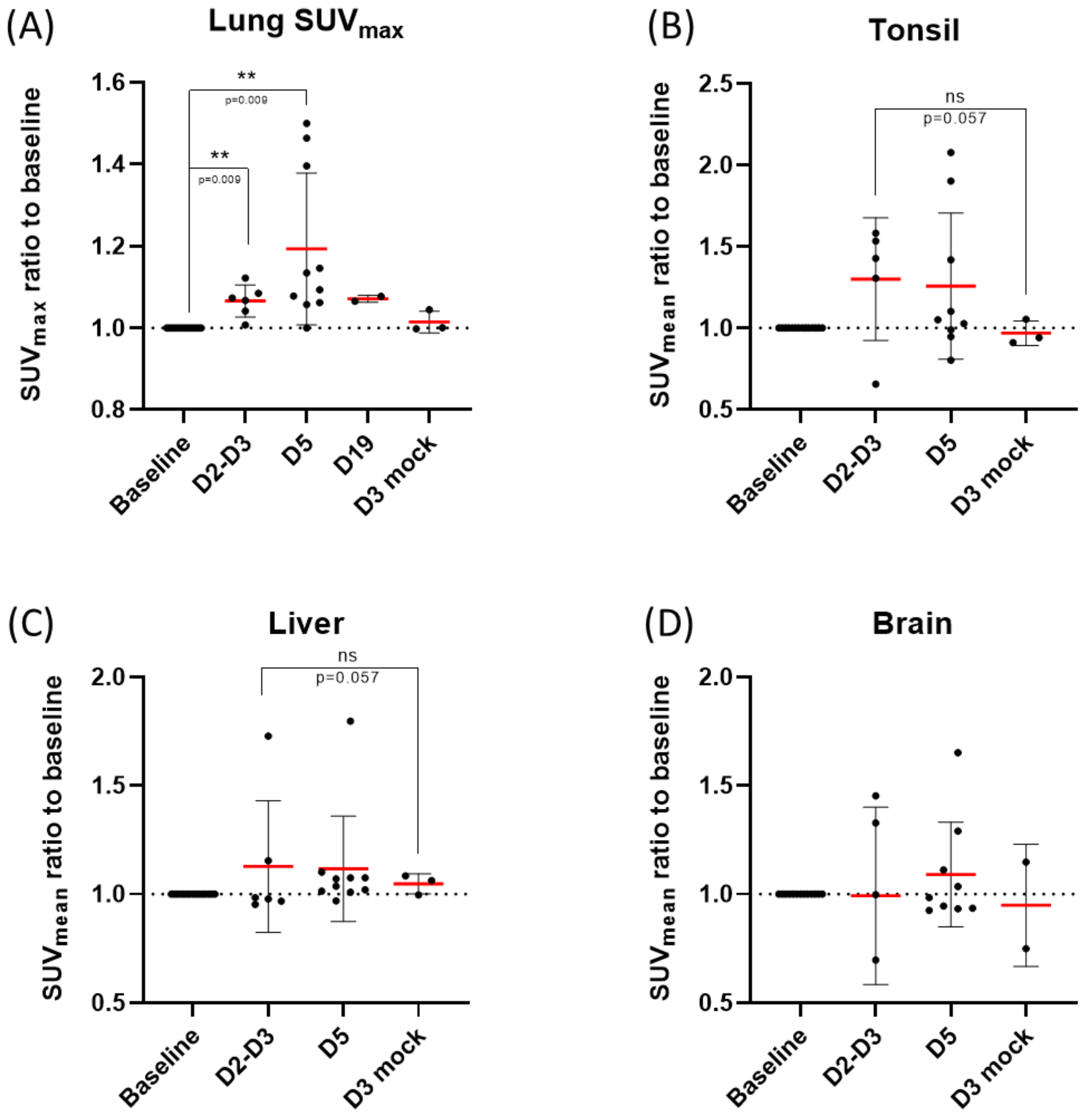

Figure S5 related to figure 6. Quantitative FDG-PET analysis of the lungs, tonsils, brain, and liver of SARS-CoV-2-exposed cynomolgus macaques. Maximum (SUV_{max}) or Mean standard uptake values (SUV_{mean}) expressed as individual ratio to baseline values for the lungs (A) , tonsils (B), liver (C) and brain (D) over time. (A) Paired t-tests (**: $p < 0.01$), (C-D) Mann-Whitney t-tests performed between D3 infected and mock-infected groups. ns: Non-significant.

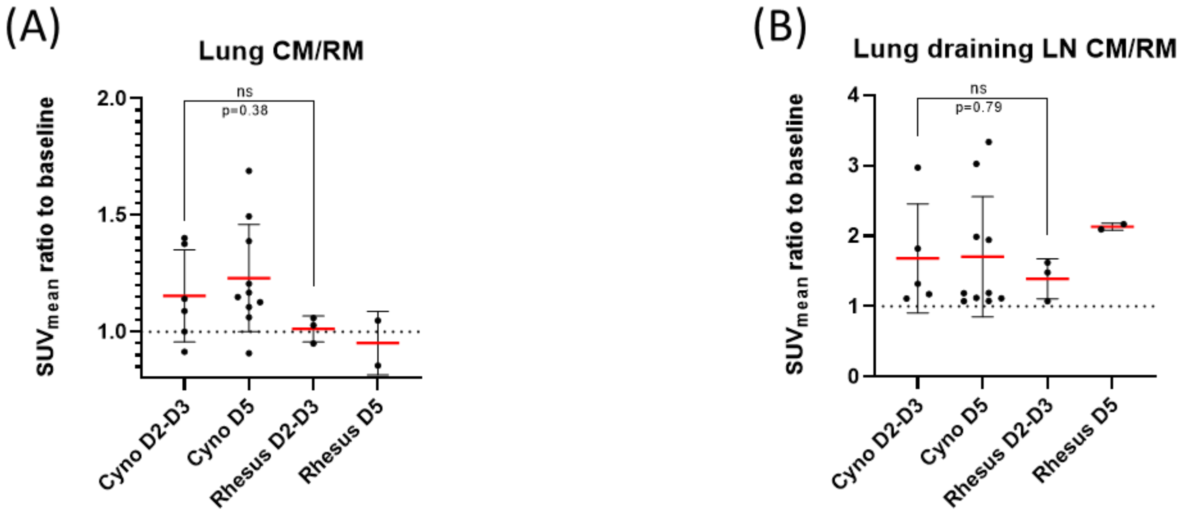

Figure S6, related to Figure 6. Comparison of the FDG PET signal for the lungs and lung-draining lymph nodes of SARS-CoV-2-exposed cynomolgus and rhesus macaques. Mean standard uptake values (SUV_{mean}) expressed as individual ratio to baseline values for the lungs (A) and lung-draining lymph nodes (B) over time. ns: non-statistically significant, Mann-Whitney t-tests.

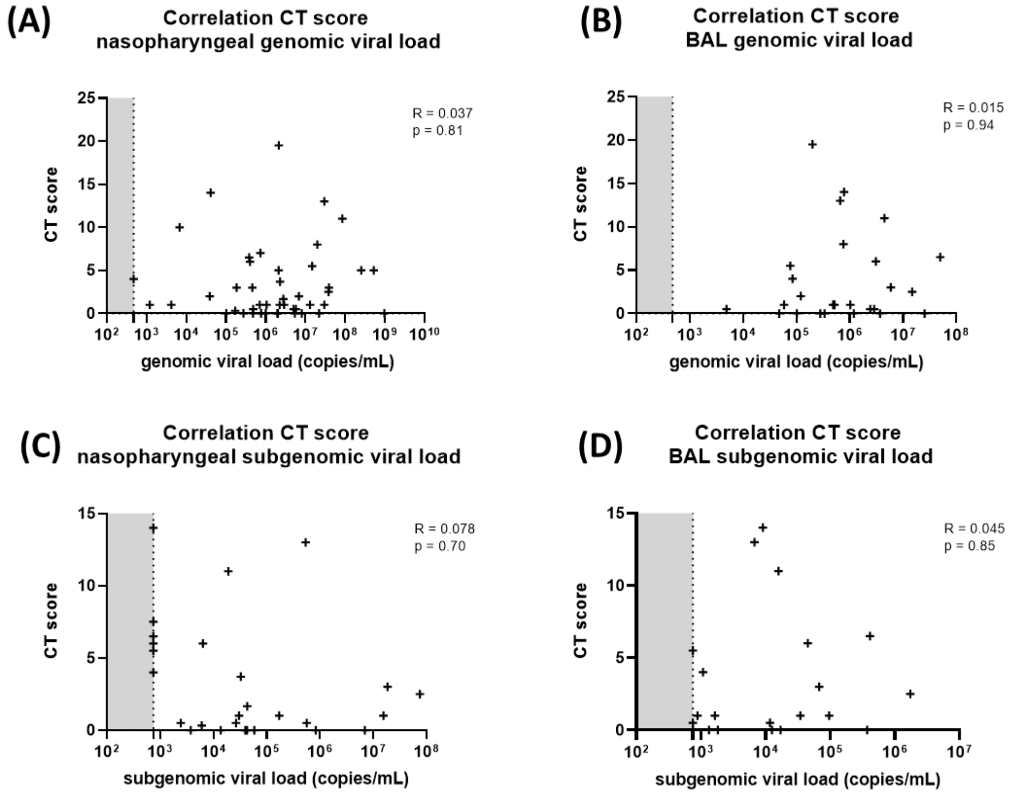

Figure S7, related to Figure 1 and Figure 3. Correlations between lung CT scores and viral loads in cynomolgus macaques. Individual nasopharyngeal (A, C) and broncho-alveolar lavage (B, D) viral loads associated with the corresponding CT-score obtained the same day (or the previous one). Titres are presented as the number of copies of genomic (A-B) and subgenomic (C-D) RNA/mL. Dotted line: limit of quantification.

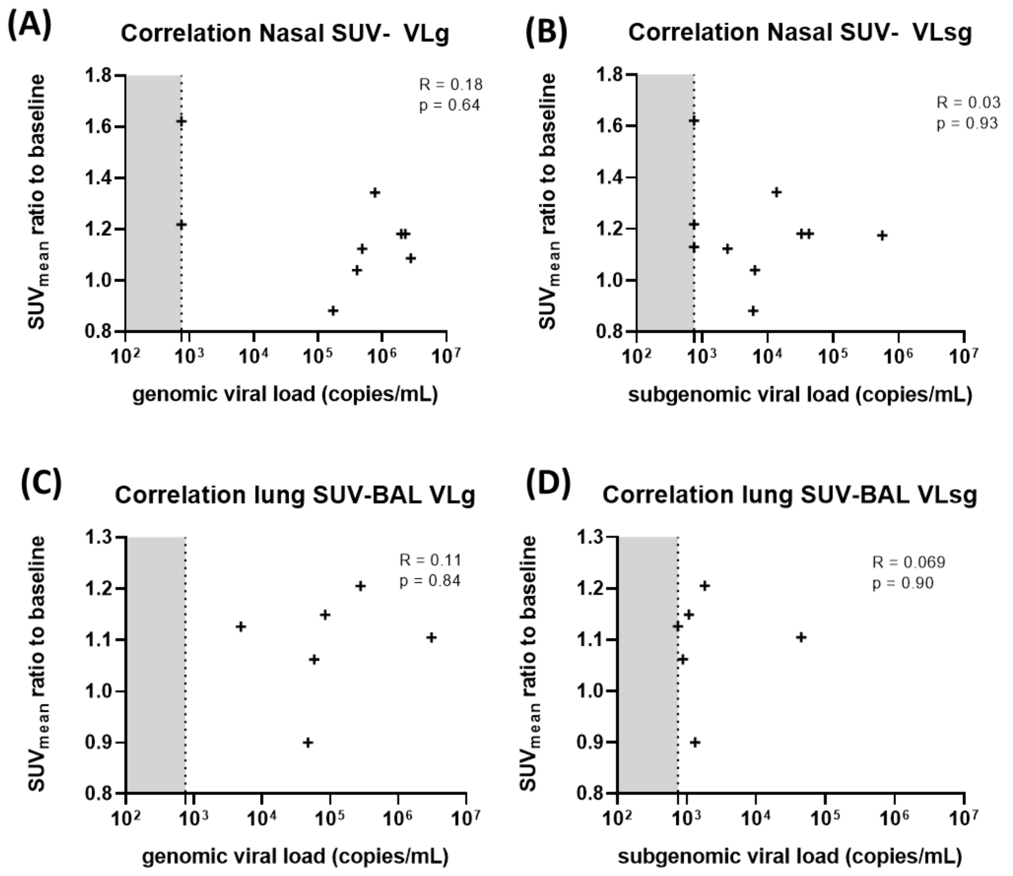

Figure S8, related to Figure 3 and Figure 6. Correlations between [^{18}F]-FDG uptake in the nasal cavity or lungs and associated local viral loads in cynomolgus macaques. Individual nasopharyngeal (A-B) and broncho-alveolar lavage (C- D) viral loads associated with the corresponding [^{18}F]-FDG mean SUV obtained the same day for the nose (A-B) and lungs (C-D). Viral loads are presented as the number of copies of genomic (A, C) and subgenomic (B, D) RNA per mL. Dotted line: limit of quantification.

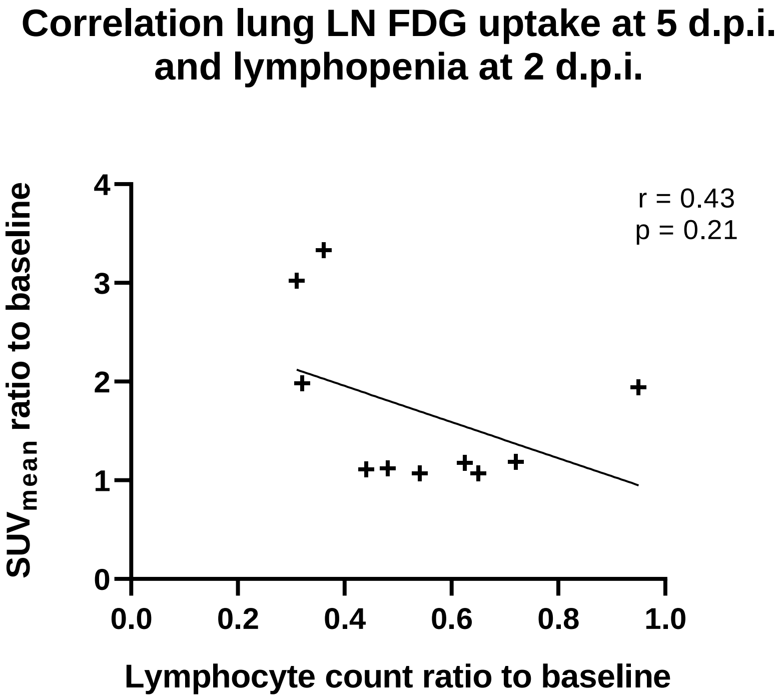

Figure S9, related to Figure 1 and Figure 6. Correlations between $[^{18}F]$-FDG uptake in the lung-draining lymph nodes at 5 d.p.i. and associated blood lymphocyte decrease at 2 d.p.i. in cynomolgus macaques.
